# Evaluation of meniscus extrusion during stair ambulation in healthy volunteers using dynamic ultrasonography: a feasibility study

**DOI:** 10.1007/s10396-023-01348-1

**Published:** 2023-08-11

**Authors:** Takato Hashizume, Yosuke Ishii, Yuko Nakashima, Saeko Okamoto, Yoshitaka Iwamoto, Kaoru Okada, Kazuya Takagi, Nobuo Adachi, Makoto Takahashi

**Affiliations:** 1 Department of Biomechanics, Graduate School of Biomedical and Health Sciences, Hiroshima University, 1-2-3 Kasumi, Minami-ku, Hiroshima, 734-8553 Japan; 2 Department of Orthopaedic Surgery, Graduate School of Biomedical & Health Sciences, Hiroshima University, Hiroshima, Japan; 3 Ultrasound Business Operations, Healthcare Business Headquarters, Konica Minolta, Inc, Tokyo, Japan

**Keywords:** Medial meniscus extrusion, Dynamic ultrasonography, Motion analysis, Stair ambulation

## Abstract

**Purpose:**

This study aimed to investigate the feasibility of evaluating medial meniscus extrusion (MME) during stair ambulation.

**Methods:**

Twenty healthy young participants (mean age, 22.4 ± 0.9 years) were recruited for this cross-sectional study. Synchronization between the three-dimensional motion system and ultrasonography was used to quantify the extent of meniscal extrusion and knee angles during different tasks, including gait, stair ascent, and stair descent. In particular, ultrasonography was used to record the movements of both the middle and posterior segments of the meniscus to obtain detailed information about these movements in relation to the knee angle. The difference between the maximum MME and the MME at the initial contact (ΔMME) was evaluated during each task in the stance phase.

**Results:**

Visualization of the meniscus in the middle segment was limited with increasing knee flexion angle, whereas the posterior segments were visible during all tasks. ΔMME of the posterior segment during stair ascent and descent was higher than that during gait (gait: 0.68 ± 0.20 mm, ascent: 1.00 ± 0.39 mm, descent: 0.90 ± 0.27 mm, gait-ascent: p = 0.009, gait-descent: p = 0.004).

**Conclusions:**

Evaluation that includes the posterior segment enables visualization of the medial meniscus and detection of its specific behavior during stair ambulation. These findings demonstrate the feasibility of evaluating meniscus dynamics during stair ambulation, and could contribute to a better understanding of these dynamics.

## Introduction

The medial meniscus absorbs and distinguishes knee joint loading during daily activities [1, 2]. However, medial meniscus extrusion (MME) causes the loss of these functions [3]. MME expands with knee joint loading [4–6], and this increase in MME leads to the progression of knee osteoarthritis (OA), particularly in the early stages [4, 5]. Therefore, prevention of initial knee OA requires an established evaluation that can sensitively detect an increase in MME during loading stress.

Previous studies have reported an increase in MME during gait, which correlates with loading stress [7, 8]. These results indicate that an increase in MME during motion under loading stress is a useful indicator for understanding the pathology of knee OA. In contrast, stair ambulation is a frequent activity in daily life and is known to produce higher knee joint loading than gait [9–11]. Therefore, the increase in MME during stair ambulation may be greater than that during gait. Additionally, an increase in MME is correlated with knee pain in daily activities [12, 13], which is critical for diagnosing early-stage knee OA [14]. Moreover, patients with knee OA experience pain during stair ambulation at an earlier stage than during gait [15]. Therefore, evaluating the increase in MME during stair ambulation could contribute to the detection of abnormal extrusion and elucidate the mechanism of knee pain in patients with early-stage knee OA. However, there is currently no established method for evaluating the increase in MME during stair ambulation.

This unclear background may be due to the kinematics of the meniscus. Typically, the meniscus translates posteriorly and intraarticularly during knee flexion [16–18]. However, a previous study reported that visualizing the meniscus during the swing phase of the gait cycle is difficult [7]. This suggests that the swing phase requires deeper knee flexion than the stance phase, and increased knee flexion causes missing meniscal images, limiting the visualization of the middle segment of the meniscus. Similarly, stair ambulation also requires deep knee flexion [9], making it challenging to use the conventional method [4–8] that only evaluates the middle segment of the entire meniscus. However, a recent study evaluated MME in the posterior segment and showed that meniscus translation in this segment was smaller than that in the middle segment [19]. This finding may enable us to obtain a minimum number of missing meniscal images and to evaluate the increase in MME during stair ambulation. Therefore, this study aimed to investigate the feasibility of evaluating MME during stair ambulation using motion analysis and ultrasound imaging, focusing on the posterior segment.

## Methods

### Participants

Twenty healthy young participants were recruited (mean age, 22.4 ± 0.9 years; BMI, 21.3 ± 2.3 kg/m^2^; female, n = 10). The exclusion criteria were a history of surgical treatment of the knee, trauma to the lower extremities, and neurological disorders. This study was approved by the institutional review board (E-2498-1) and was conducted in accordance with the Declaration of Helsinki. All participants provided appropriate informed consent to participate in this study.

### Motion analysis

**Fig. 1:**
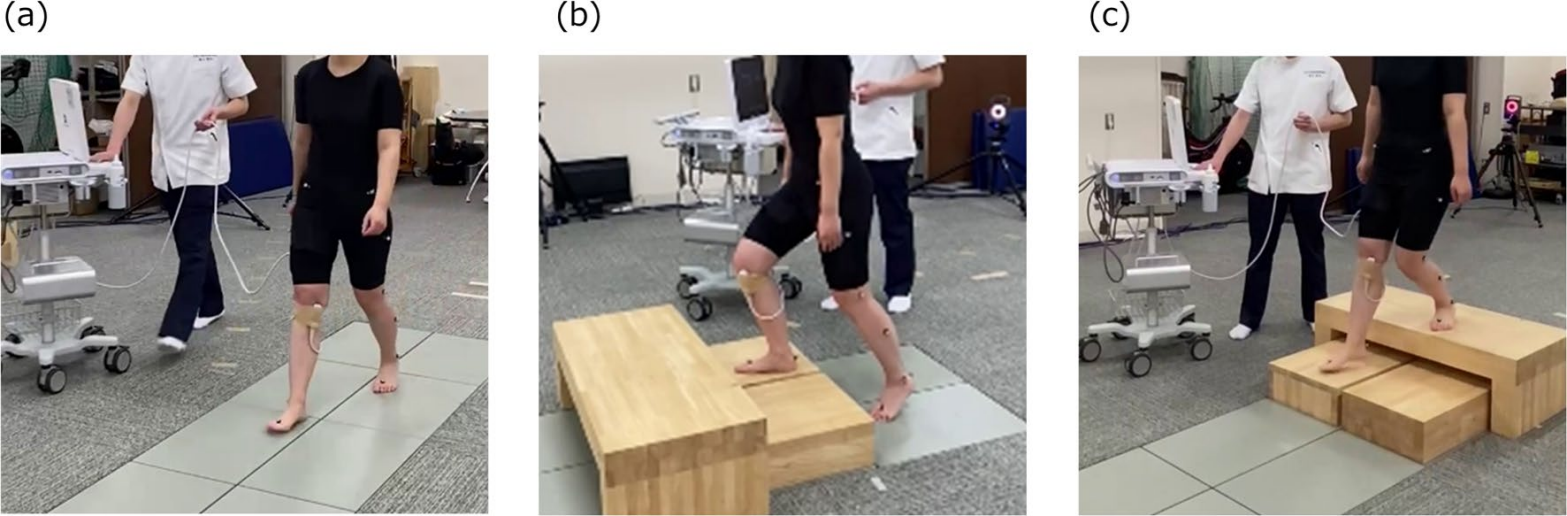
Snapshot of motion analysis during gait (**a**), stair ascent (**b**), and stair descent (**c**) tasks

Knee flexion angles during gait, stair ascent, and stair descent were obtained at a frequency of 100 Hz using a three-dimensional motion analysis system (Vicon Motion Systems, Oxford, UK) equipped with 16 cameras (Vicon Motion Systems, Oxford, UK). Eight force plates (AMTI, Watertown, MA, USA) were synchronized with the cameras, and the vertical ground reaction force (GRF) was measured at a sampling frequency of 1000 Hz. In this study, a Vicon Plug-in Gait lower body model was adopted, and 16 passive reflective markers were placed on the landmarks of each participant’s body. The experimental procedure involved the examiner first determining the axes of each participant’s joints in a static position, after which the participants were instructed to walk 5 m, ascend, and descend the stairs at a comfortable speed (Fig. 1). The height of the stairs was 30 cm, and the participants performed all tasks without any external support. Each task was repeated thrice, and the analysis interval was the stance phase, defined as the period from the initial contact to toe-off during the task. These events were identified using a 20-N threshold on the GRF.

The knee flexion range of motion and GRF peaks were calculated as biomechanical data. The GRF has bi-peak and detects the first and second peaks. A previous study showed that an increase in MME during gait correlated with GRF [8]; therefore, this value was used as the representative loading stress in this study.

### Medial meniscus extrusion evaluation

**Fig. 2:**
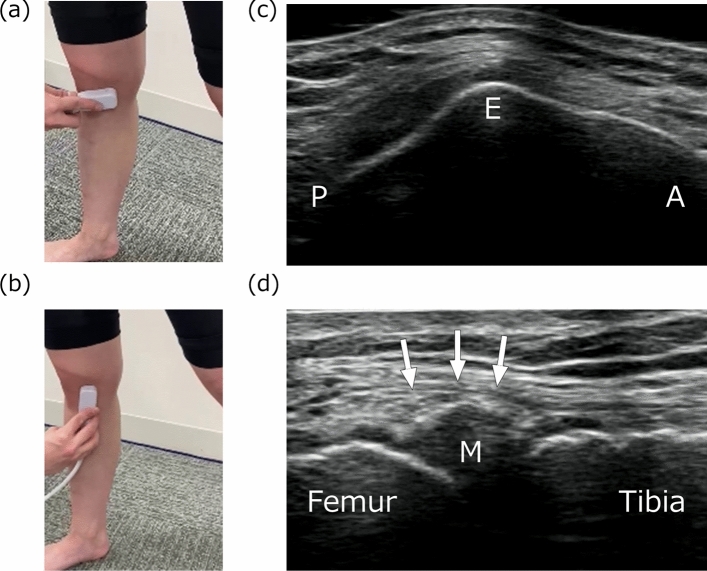
Procedure for evaluation of the middle segment of the medial meniscus. Setting position of the transducer (**a**, **b**) and representative ultrasound images of the medial epicondyle of femur (**c**) and middle segment of the medial meniscus (**d**). *E* medial epicondyle of femur, *M* medial meniscus, *Arrow* medial collateral ligament, *P* posterior, *A* anterior

**Fig. 3:**
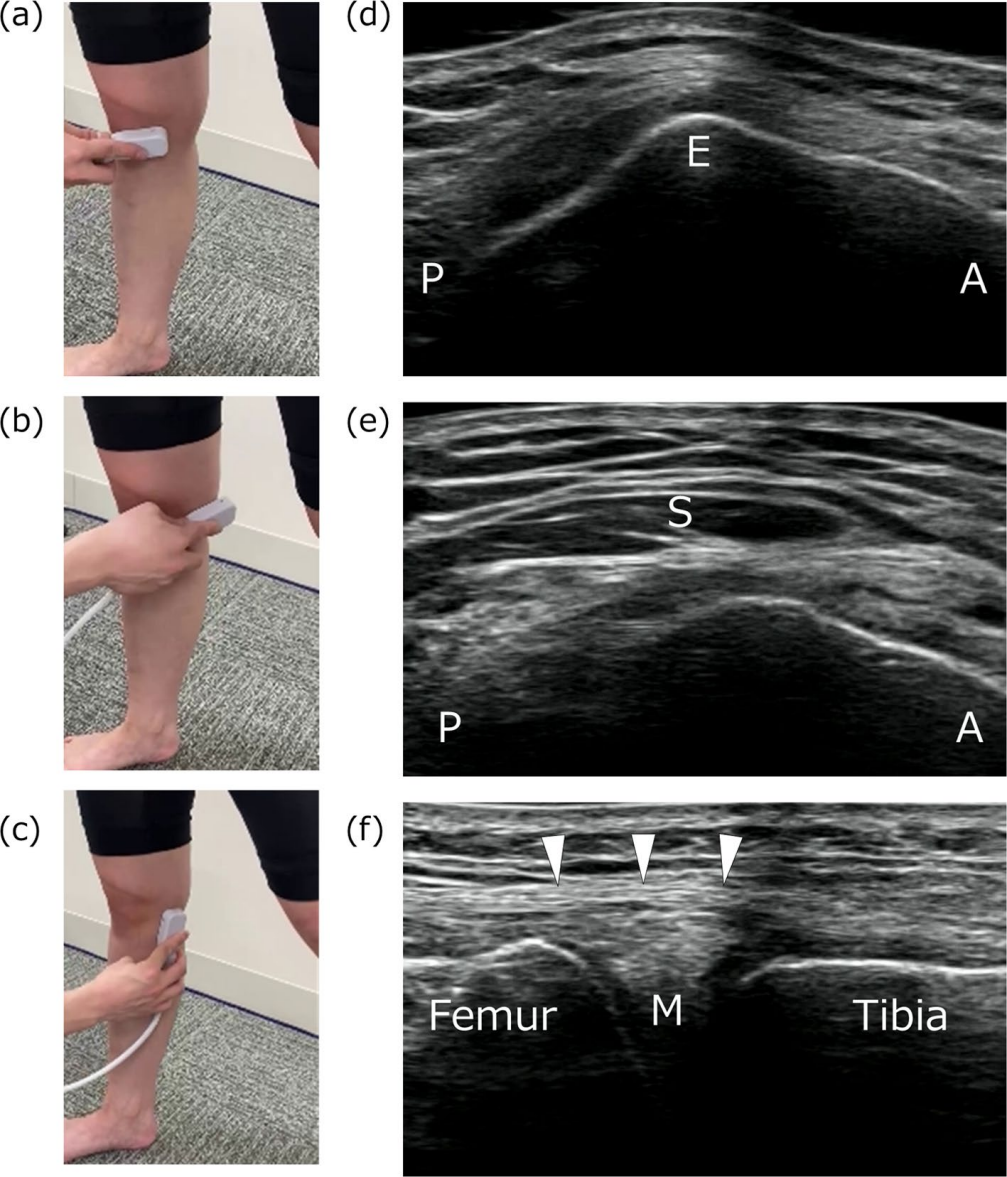
Procedure for evaluation of the posterior segment of the medial meniscus. Setting position of the transducer (**a**, **b**, **c**) and representative ultrasound images of the medial epicondyle of femur (**d**), sartorius (**e**), and posterior segment of the medial meniscus (**f**). *E* medial epicondyle of femur, *M* medial meniscus, *S* sartorius, *Arrowheads*, posterior oblique ligament; *P*, posterior; *A*, anterior

Ultrasound imaging of the medial meniscus during each task was performed using an ultrasound device (SNiBLE; KONICA MINOLTA, Japan) with a prototype 3-11-MHz special linear- array transducer (KONICA MINOLTA, Japan). A longitudinal transducer was fitted onto the medial surface of the knee. In this study, meniscal images in both the middle and posterior segments were obtained to apply meniscus movement to the medial compartment based on previous studies [7, 8]. This posterior segment indicates the posterior part of the meniscus body but not the posterior horn. The following procedure was used to obtain clear images of the middle segment (Fig. 2). First, the top notch on the medial epicondyle of femur was depicted as the landmark on ultrasound, and then a vertical line passing through the medial joint line was drawn from the medial epicondyle of femur to the proximal tibia. Second, the transducer was adjusted and fixed to obtain a clear image of the meniscus and the medial collateral ligament along the vertical line. To evaluate the posterior segment of the meniscus (Fig. 3), the top notch on the medial epicondyle of femur was first identified, and the transducer was moved posteriorly to visualize the sartorius in the center of the image. Then, a vertical line was drawn from the setting point to the proximal tibia, passing through the medial joint line. Finally, the transducer was adjusted and fixed to obtain a clear image of the meniscus along a vertical line. The sartorius functions in knee flexion and is located posteriorly to the medial epicondyle of femur. Sartorius location was used as a landmark to determine the posterior segment of the medial meniscus. Additionally, the posterior oblique ligament was observed as a secondary landmark during tasks in which the posterior segment of the meniscus was visualized. This ligament is located posterior to the medial collateral ligament [20–22]. Therefore, the examiner checked whether the posterior oblique ligament was stably visualized in order to confirm the quality of the images obtained during the tasks.

Ultrasound imaging was performed at 30 Hz for all the tasks. Analog signals were sent simultaneously from out-device to synchronize the motion capture system and ultrasonography, and the starting time between these devices was determined. These analyses were performed using the Kinovea software (v0.8.15; Kinovea Open Source Project, https://www.kinovea.org).

The MME value was defined as the distance between the medial tibial plateau cortex and the outermost edge of the medial meniscus [23]. Approximately 25 and 30 ultrasound images were acquired during gait and stair ambulation, respectively. In addition, the increase in MME was defined as ΔMME using the difference between the maximum MME and MME at the initial contact. For these data, the average value for three trials was used for statistical analysis and time-normalized to 101 points of data according to the stance phase of the gait cycle.

To evaluate the reliability of ΔMME, intra- and inter-rater reliabilities were analyzed using the intraclass correlation coefficient (ICC). The two examiners obtained additional data from six participants twice over three days. One examiner (T.H.) obtained ICC (1, 3), and ICC (2, 3) was obtained by T.H. and another examiner (Y.I.) who was blinded to the participant’s information. The ICC values were interpreted as follows: ICC < 0.50, poor reliability; 0.50 < ICC < 0.75, moderate reliability; 0.75 < ICC < 0.90, good reliability; 0.90 < ICC, excellent reliability [24].

### Statistical analysis

All statistical analyses were performed using SPSS (v23, IBM, USA). The data are expressed as mean (standard deviation). The normality of ΔMME was confirmed using Shapiro–Wilk test. Repeated measures analysis of variance was performed to compare the data between the three tasks (gait, ascent, descent), with Bonferroni corrections used for post-hoc tests. In addition, Pearson or Spearman correlation analyses were performed to investigate the correlation between ΔMME and GRF peaks or ΔMME itself among the tasks. The significance level was set at 5%.

## Results

### Reliability of MME measurements

The reliability of the ΔMME measurements during gait and stair ambulation are listed in Table 1. All intra- and inter-rater reliabilities were found to have good to excellent levels of reliability.

**Table 1:** Reliability of ΔMME measurements during tasks

	ICC (1, 3)	p	ICC (2, 3)	p
Middle segment during gait	0.949 (0.695–0.993)	0.001	0.879 (0.273–0.983)	0.012
Posterior segment during gait	0.889 (0.333–0.984)	0.009	0.969 (0.813–0.996)	0.000
Posterior segment during ascent	0.962 (0.773–0.995)	0.001	0.884 (0.304–0.983)	0.010
Posterior segment during descent	0.902 (0.411–0.986)	0.007	0.946 (0.676–0.992)	0.001

The values in parentheses represent the 95% confidence interval for ICC

*ICC* intraclass correlation coefficient, *MME* medial meniscus extrusion

### Evaluation of MME of the middle and posterior segments

**Fig. 4:**
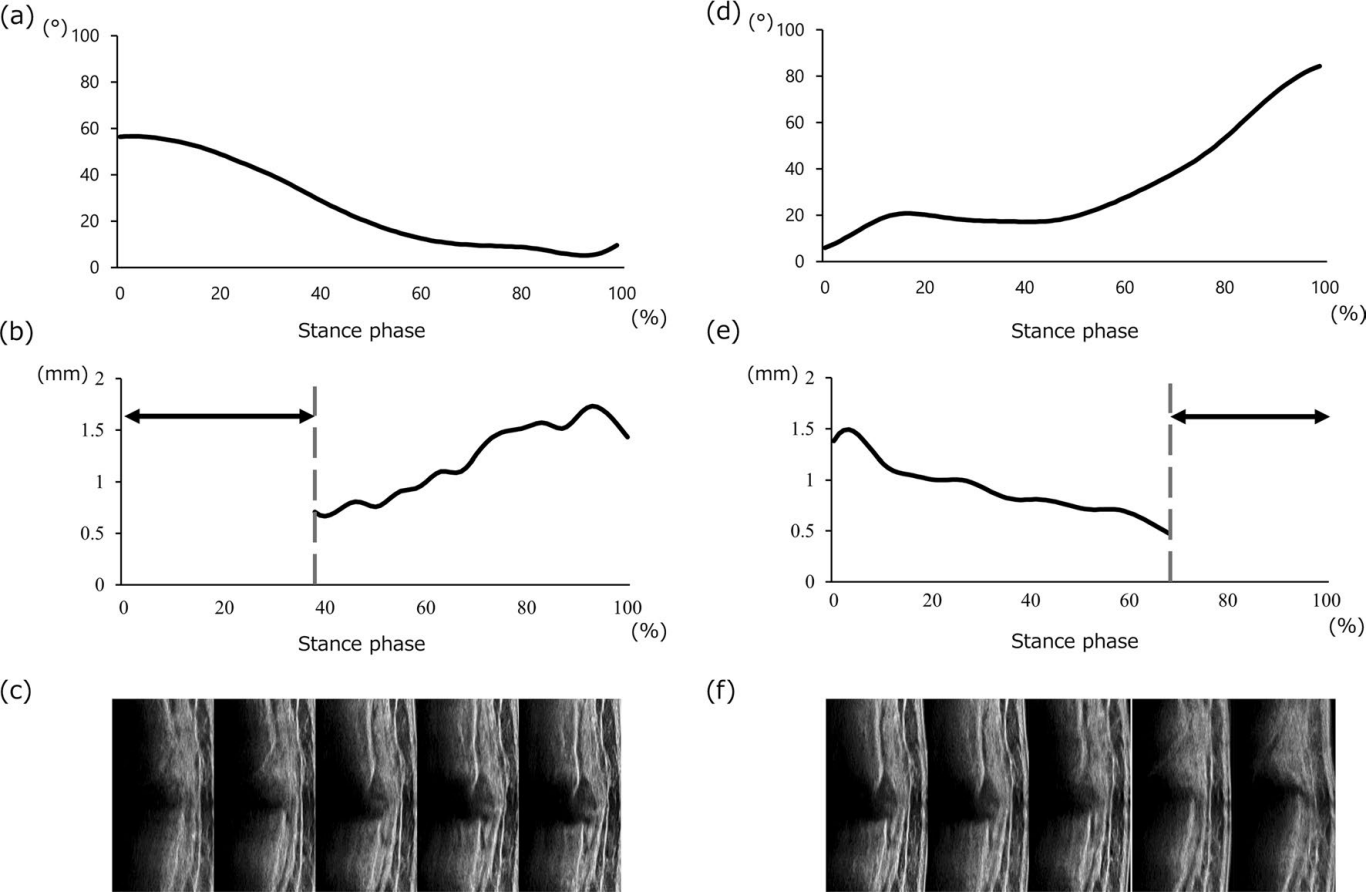
Representative knee flexion angle, the waveform of MME, and images of the middle segment of the medial meniscus during stair ascent (**a**, **b**, **c**) and descent (**d**, **e**, **f**) in one participant. Double-headed arrows show the interval with compromised meniscus evaluation

**Fig. 5:**
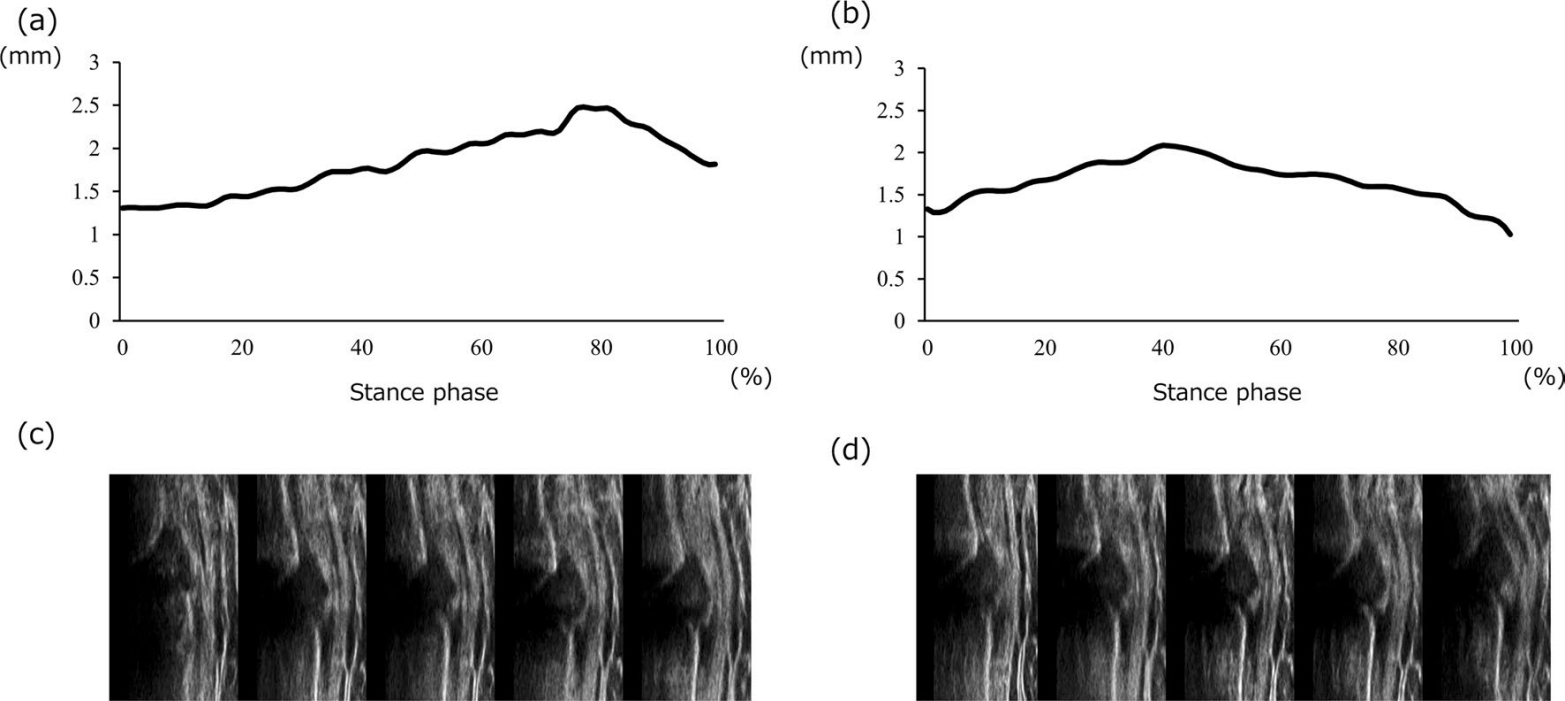
Representative waveform of MME and images of the posterior segment of the medial meniscus while ascending (**a**, **c**) and descending (**b**, **d**) stairs in one participant

During the analysis interval of the gait task, the meniscus was visualized in the middle segment. However, during both ascending and descending tasks, the visibility of the outermost edge of the meniscus was compromised in 10 (50%) out of 20 and 17 (85%) out of 20 participants, respectively. Specifically, while ascending the stairs, it was difficult to visualize a clear edge between the initial contact and 35.2% (15.5%) of the stance phase, and the knee flexion angle at the point where the edge could be visualized was 41.46° (12.81°) (Fig. 4a, b, c). Conversely, while descending the stairs, the clear edge was difficult to visualize after 61.3% (21.4%) of the stance phase, and the flexion angle at the point where the edge could be visualized was 37.17° (14.61°) (Fig. 4d, e, f). In the posterior segment, the outermost edge of the meniscus could be visualized during all tasks, including those performed at different knee flexion angles (Fig. 5).

### Comparison and correlation of MME and ΔMME among tasks

**Fig. 6:**
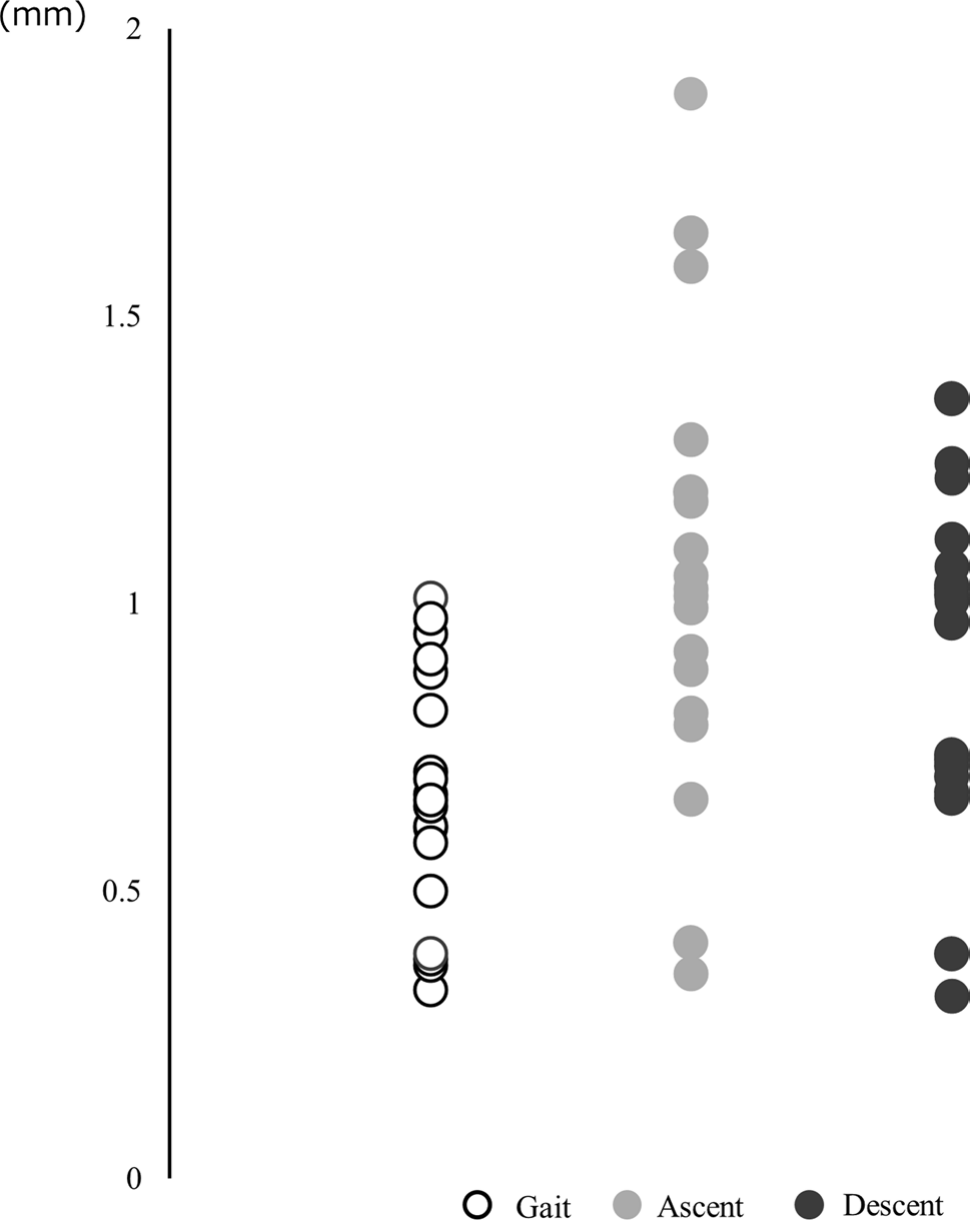
Scatter plot of ΔMME during gait, stair ascent, and stair descent

No difference was observed between ΔMME of the middle and posterior segments during gait [middle: 0.67 mm (0.24 mm), posterior: 0.68 mm (0.20 mm), p = 0.90]. The MME data for the posterior segment are presented in Table 2. The maximum MME and ΔMME during stair ambulation were higher than during gait. In addition, the dispersion of ΔMME during stair ambulation tended to be larger than that during gait (Fig. 6). There was no correlation between ΔMME among the tasks (gait—ascent: r = 0.08, p = 0.74; gait—descent: r = 0.41, p = 0.07; ascent—descent: r = −0.06, p = 0.81).

**Table 2:** Value of posterior segment of MME during tasks

	Gait	Ascent	Descent
Minimum MME (mm)	0.45 ± 0.94^†^	0.34 ± 0.85	0.20 ± 1.09
Maximum MME (mm)	1.20 ± 0.93	1.53 ± 0.82^*^	1.53 ± 0.94^††^
MME at initial contact (mm)	0.52 ± 0.96	0.63 ± 1.04	0.52 ± 0.87
ΔMME (mm)	0.68 ± 0.20	1.00 ± 0.39^**^	0.90 ± 0.27^††^

The values represent the mean ± standard deviation. *MME* medial meniscus extrusion

*Difference between gait and stair ascent using the post-hoc test (p < 0.05)

**Difference between gait and stair ascent using the post-hoc test (p < 0.01)

^†^Difference between gait and stair descent using the post-hoc test (p < 0.05)

^††^Difference between gait and stair descent using the post-hoc test (p < 0.01)

### Biomechanical data and correlation between ΔMME and GRF peaks

Knee flexion angles and GRF peaks are shown in Table 3. The knee flexion angle during stair ambulation was higher than that during gait. During stair ascent, the knee flexion angle decreased during the stance phase (Fig. 4a). In contrast, during stair descent, the knee flexion angle increased during the stance phase (Fig. 4d). The first and second GRF peaks during stair ascent and descent, respectively, were higher than those during the gait. No correlation was observed between ΔMME and GRF peaks (Table 4).

**Table 3:** Knee flexion range of motion and GRF peaks during tasks

	Gait	Ascent	Descent
Knee flexion angle (°)	34.12 ± 5.75	55.68 ± 6.20^*^	77.75 ± 5.67^*‡^
GRF first peak (N)	594.41 ± 128.87	594.98 ± 112.12	759.20 ± 153.08^*‡^
GRF second peak (N)	613.76 ± 118.66^†^	672.31 ± 111.80^*‡^	562.68 ± 114.59

Values represent the mean ± standard deviation

*GRF* ground reaction force

*Difference between gait and stair ascent using the post-hoc test (p < 0.05)

^†^Difference between gait and stair descent using the post-hoc test (p < 0.05)

^‡^Difference between stair ascent and descent using the post-hoc test (p < 0.05)

**Table 4:** Correlation between ΔMME and GRF peaks during tasks

	Gait		Ascent		Descent	
	r	p	r	p	r	p
GRF first peak	−0.33	0.16	−0.27	0.25	−0.09	0.46
GRF second peak	−0.18	0.44	0.05	0.29	−0.25	0.63

The r values represent the correlation coefficients between ΔMME and GRF peaks during tasks. *MME* medial meniscus extrusion, *GRF* ground reaction force

## Discussion

This is the first study to evaluate MME during stair ambulation. The middle segment of the medial meniscus was difficult to visualize in some cases due to the increased knee flexion. In contrast, the posterior segment of the meniscus was visualized in all participants, indicating the feasibility of MME evaluation during stair ambulation.

Visualization of the outermost edge in the middle segment of the meniscus during gait was possible; however, this can be difficult during stair ambulation in some cases. In a previous study [7], it was challenging to visualize the middle segment of the medial meniscus during the swing phase of the gait cycle when the knee flexion angle was increased. The knee flexion angles in the initial swing phase of the gait cycle are approximately 40° [25, 26]. In this study, the knee flexion angle at which the outermost edge of the middle segment was difficult to visualize during stair ambulation were 41.46° (12.81°) and 37.17° (14.61°). These findings were consistent with previous studies [25, 26]. In general, the meniscus moves posteriorly as the knee flexes [16–18]. These studies suggested that the appropriate reach of the ultrasound wave into the meniscus might be restricted by this movement, resulting in unclear visibility of the outermost edge of the medial meniscus during motion. Therefore, the conventional method of focusing on the middle segment [7, 8] may explain the difficulty encountered in visualizing the outermost edge of the meniscus when the knee flexion angle exceeds a certain value.

On the other hand, the posterior segment of the meniscus was visualized during the stance phase in all the tasks. In a previous study, the MME value of the middle segment during knee flexion was lower than that during knee extension, but this was not observed in the posterior segment [19]. This suggests that the posterior segment undergoes less intra-articular translation of the meniscus with knee flexion compared to the middle segment. In this study, the examiner placed a transducer on the posterior segment to visualize the meniscus during stair ambulation. Our results showed a good or excellent ICC, and the posterior oblique ligament was consistently visible at the same location throughout the tasks. Therefore, our findings, along with those of previous studies, suggest that current techniques are suitable for evaluating MME during stair ambulation and can provide a detailed understanding of meniscal dynamics.

In the present study, ΔMME and GRF peaks during stair ambulation were higher than those during gait, but there was no correlation between them. In a previous study, knee joint loading was correlated with the ΔMME value [8], but this was not observed in our study. Stair ambulation requires more knee flexion than gait [9, 25]. The present study also showed a tendency toward decreased MME during stair ambulation at high flexion angles. The meniscus undergoes both posterior and intra-articular translation with knee flexion [18, 27, 28], which may obscure the effect of knee joint loading on ΔMME during stair ambulation. Therefore, our findings suggest that to understand the mechanism of ΔMME, it is necessary to consider not only knee joint loading but also the knee flexion angle.

In addition, there was no correlation between ΔMME and gait or stair ambulation. This indicates that ΔMME during gait may not be able to predict those during stair ambulation as the movement of the meniscus is independent of each motion. In patients with early knee OA, changes in MME from 0° to 90° knee flexion in static evaluation may be associated with meniscal lesions [29], and ΔMME occurring under loading stress in any situation is known to reflect the pain and pathology in the early stages of knee OA [4, 5, 12, 13, 30]. Therefore, it is important to develop an established method for evaluating ΔMME. A recent study demonstrated that ΔMME in dynamic evaluation was higher than that in static evaluation and accurately reflected knee pain [31]. Most patients with knee OA experience knee pain during stair ambulation at an earlier stage than gait [15]. Additionally, in this study, evaluation of ΔMME showed greater dispersion during stair ambulation than during gait. These findings may contribute to the understanding of the individual mechanisms of pain and mechano-pathology, and help identify patients with knee OA from an earlier stage.

The present study had several limitations. First, it aimed to investigate the feasibility of evaluating MME during stair ambulation. Therefore, reproducibility and validity were not given sufficient consideration. In this study, evaluation of MME in the posterior segment did not include the bony landmark, which could have caused an error in ΔMME. Further investigation is required to obtain high reproducibility and validity by employing a rigorous methodology. Second, continuous stair ambulation was not performed, which could have resulted in underestimation of ΔMME in activities of daily living. A more comprehensive investigation involving continuous stair ambulation is recommended for future studies. Third, the underlying reason for visualizing the outermost edge of the middle segment during stair ambulation in some participants could not be determined. Previous studies have suggested that factors such as tibial rotation, meniscus size, and morphology of the medial epicondyle of femur may be associated with meniscal movement [17, 32, 33]. Further investigation of the association between these factors and meniscal movement is necessary to better understand this phenomenon. Finally, this study did not include patients with knee OA; therefore, the results may not be generalizable to this population. Future studies should include patients with knee OA and incorporate morphological and clinical information in natural situations during stair ambulation to enhance our understanding of the meniscal dynamics in this population.

## Conclusion

Ultrasonographic evaluation of the posterior segment enables visualization of the medial meniscus and detection of its specific behavior during stair ambulation. The findings from our study demonstrated the feasibility of evaluating meniscal dynamics during stair ambulation and contributed to a better understanding of these dynamics.

## Data Availability

Not applicable.

## References

[1] Walker PS, Arno S, Bell C (2015). Function of the medial meniscus in force transmission and stability. J Biomech.

[2] Jones RS, Keene G, Learmonth D (1996). Direct measurement of hoop strains in the intact and torn human medial meniscus. Clin Biomech.

[3] Gokkus K, Atmaca H, Uğur L (2016). The relationship between medial meniscal subluxation and stress distribution pattern of the knee joint: finite element analysis. J Orthop Sci.

[4] Kawaguchi K, Enokida M, Otsuki R (2012). Ultrasonographic evaluation of medial radial displacement of the medial meniscus in knee osteoarthritis. Arthritis Rheum.

[5] Murakami T, Enokida M, Kawaguchi K (2017). Useful ultrasonographic evaluation of the medial meniscus as a feature predicting the onset of radiographic knee osteoarthritis. J Orthop Sci.

[6] Achtnich A, Petersen W, Willinger L (2018). Medial meniscus extrusion increases with age and BMI and is depending on different loading conditions. Knee Surg Sports Traumatol Arthrosc.

[7] Ishii Y, Nakashima Y, Ishikawa M (2020). Dynamic ultrasonography of the medial meniscus during walking in knee osteoarthritis. Knee.

[8] Ishii Y, Ishikawa M, Nakashima Y (2022). Knee adduction moment is correlated with the increase in medial meniscus extrusion by dynamic ultrasound in knee osteoarthritis. Knee.

[9] Nadeau S, McFadyen BJ, Malouin F (2003). Frontal and sagittal plane analyses of the stair climbing task in healthy adults aged over 40 years: what are the challenges compared to level walking?. Clin Biomech.

[10] Liikavainio T, Isolehto J, Helminen HJ (2007). Loading and gait symmetry during level and stair walking in asymptomatic subjects with knee osteoarthritis: Importance of quadriceps femoris in reducing impact force during heel strike?. Knee.

[11] Kutzner I, Heinlein B, Graichen F (2010). Loading of the knee joint during activities of daily living measured in vivo in five subjects. J Biomech.

[12] Ishii Y, Ishikawa M, Nakashima Y (2021). Association between medial meniscus extrusion under weight-bearing conditions and pain in early-stage knee osteoarthritis. J Med Ultrason.

[13] Ishii Y, Ishikawa M, Nakashima Y (2022). Dynamic response of medial meniscus extrusion to the lateral wedge insole is correlated with immediate pain reduction in knee osteoarthritis patients: real-time ultrasonographic study. J Med Ultrason.

[14] Madry H, Kon E, Condello V (2016). Early osteoarthritis of the knee. Knee Surg Sports Traumatol Arthrosc.

[15] Hensor EMA, Dube B, Kingsbury SR (2015). Toward a clinical definition of early osteoarthritis: onset of patient-reported knee pain begins on stairs. Data from the osteoarthritis initiative. Arthritis Care Res.

[16] Kim E, Kim YJ, Cha JG (2015). Kinematic change of the meniscus and the tibiofemoral joint space in asymptomatic volunteers using a wide bore 3T closed MRI system. Skeletal Radiol.

[17] Yao J, Lancianese SL, Hovinga KR (2008). Magnetic resonance image analysis of meniscal translation and tibio-menisco-femoral contact in deep knee flexion. J Orthop Res.

[18] Scholes C, Houghton ER, Lee M (2015). Meniscal translation during knee flexion: what do we really know?. Knee Surg Sports Traumatol Arthrosc.

[19] Condron NB, Knapik DM, Gilat R (2022). Concomitant meniscotibial ligament reconstruction decreases meniscal extrusion following medial meniscus allograft transplantation: a cadaveric analysis. Arthrosc J Arthrosc Relat Surg.

[20] Vieira TD, Pioger C, Franck F (2019). Arthroscopic dissection of the distal semimembranosus tendon: an anatomical perspective on posteromedial instability and ramp lesions. Arthrosc Tech.

[21] Athwal KK, Willinger L, Shinohara S (2020). The bone attachments of the medial collateral and posterior oblique ligaments are defined anatomically and radiographically. Knee Surg Sports Traumatol Arthrosc.

[22] De Maeseneer M, Marcelis S, Boulet C (2014). Ultrasound of the knee with emphasis on the detailed anatomy of anterior, medial, and lateral structures. Skeletal Radiol.

[23] Ishii Y, Deie M, Fujita N (2017). Effects of lateral wedge insole application on medial compartment knee osteoarthritis severity evaluated by ultrasound. Knee.

[24] Koo TK, Mae YL (2016). A guideline of selecting and reporting intraclass correlation coefficients for reliability research. J Chiropr Med.

[25] Riener R, Rabuffetti M, Frigo C (2002). Stair ascent and descent at different inclinations. Gait Posture.

[26] Chou LS, Song SM, Draganich LF (1995). Predicting the kinematics and kinetics of gait based on the optimum trajectory of the swing limb. J Biomech.

[27] Chen H-N, Yang K, Dong Q-R (2014). Assessment of tibial rotation and meniscal movement using kinematic magnetic resonance imaging. J Orthop Surg.

[28] Liu T, Shen X, Ji Q (2021). The MRI-based 3D morphologic changes of knee meniscus under knee weight-bearing and early flexion conditions. Sci Rep.

[29] Shimozaki K, Nakase J, Asai K, et al. Usefulness of ultrasonography for dynamic evaluation of medial meniscus hoop function in early knee osteoarthritis. Sci Rep. 2021;11:20091. 10.1038/s41598-021-99576-3 PMC8505604 34635735

[30] Ishii Y, Ishikawa M, Kurumadani H (2020). Increase in medial meniscal extrusion in the weight-bearing position observed on ultrasonography correlates with lateral thrust in early-stage knee osteoarthritis. J Orthop Sci.

[31] Ishii Y, Ishikawa M, Nakashima Y (2023). Dynamic ultrasound reveals the specific behavior of the medial meniscus extrusion in patients with knee osteoarthritis. BMC Musculoskelet Disord.

[32] Cho JC, Tollefson L, Reckelhoff K (2021). Sonographic evaluation of the degree of medial meniscal extrusion during Thessaly test in healthy knees. Chiropr Man Ther.

[33] Boxheimer L, Lutz AM, Treiber K (2004). MR imaging of the knee: position related changes of the menisci in asymptomatic volunteers. Invest Radiol.

