# Correction: Evaluation of meniscus extrusion during stair ambulation in healthy volunteers using dynamic ultrasonography: a feasibility study

**DOI:** 10.1007/s10396-024-01432-0

**Published:** 2024-03-16

**Authors:** Takato Hashizume, Yosuke Ishii, Yuko Nakashima, Saeko Okamoto, Yoshitaka Iwamoto, Kaoru Okada, Kazuya Takagi, Nobuo Adachi, Makoto Takahashi

**Affiliations:** 1 Department of Biomechanics, Graduate School of Biomedical and Health Sciences, Hiroshima University, 1-2-3 Kasumi, Minami-ku, Hiroshima, 734-8553 Japan; 2 Department of Orthopaedic Surgery, Graduate School of Biomedical & Health Sciences, Hiroshima University, Hiroshima, Japan; 3 Ultrasound Business Operations, Healthcare Business Headquarters, Konica Minolta, Inc, Tokyo, Japan

**Correction to: Journal of Medical Ultrasonics (2023) 50:541–549**, 10.1007/s10396-023-01348-1

The article “Evaluation of meniscus extrusion during stair ambulation in healthy volunteers using dynamic ultrasonography: a feasibility study”, written by Takato Hashizume, Yosuke Ishii, Yuko Nakashima, Saeko Okamoto, Yoshitaka Iwamoto, Kaoru Okada, Kazuya Takagi, Nobuo Adachi and Makoto Takahashi, was originally published Online First without Open Access. After publication in volume 50, issue 4, page 541–549 the author decided to opt for Open Choice and to make the article an Open Access publication. Therefore, the copyright of the article has been changed to © The Author(s) 2023 and the article is forthwith distributed under the terms of the Creative Commons Attribution 4.0 International License, which permits use, sharing, adaptation, distribution and reproduction in any medium or format, as long as you give appropriate credit to the original author(s) and the source, provide a link to the Creative Commons licence, and indicate if changes were made. The images or other third party material in this article are included in the article’s Creative Commons licence, unless indicated otherwise in a credit line to the material. If material is not included in the article’s Creative Commons licence and your intended use is not permitted by statutory regulation or exceeds the permitted use, you will need to obtain permission directly from the copyright holder. To view a copy of this licence, visit http://creativecommons.org/licenses/by/4.0.

The original article has been corrected.

